# Remission of Type 2 Diabetes Mellitus (T2DM) after Sleeve Gastrectomy (SG), One-Anastomosis Gastric Bypass (OAGB), and Roux-en-Y Gastric Bypass (RYGB): A Systematic Review

**DOI:** 10.3390/medicina59050985

**Published:** 2023-05-19

**Authors:** Vignesh Balasubaramaniam, Sjaak Pouwels

**Affiliations:** 1 Department of General Surgery, Ysbyty Gwynedd Hospital, Bangor LL57 2FA, UK; vigneshb586@gmail.com; 2 Department of Intensive Care Medicine, Elisabeth-Tweesteden Hospital, P.O. Box 9051, 5000 LC Tilburg, The Netherlands; 3 Department of General, Abdominal and Minimally Invasive Surgery, Helios Klinikum, Lutherplatz 40, 47805 Krefeld, Germany

**Keywords:** type 2 diabetes mellitus, sleeve gastrectomy, Roux-en-Y gastric bypass, one-anastomosis gastric bypass, diabetes remission

## Abstract

*Background and Objectives*: The three most widely performed bariatric surgeries are Roux-en-Y gastric bypass (RYGB), sleeve gastrectomy (SG), and one-anastomosis gastric bypass (OAGB). Aside from the benefits of weight loss, current findings suggest that these procedures can also induce remission of T2DM (type 2 diabetes mellitus). There are limited data that directly compare these three procedures. This study aims to compare the short-term and long-term remission of T2DM after RYGB, SG, and OAGB. *Materials and Methods*: Three databases (Embase, PubMed, and Cochrane) were searched for randomised controlled trials, prospective studies, and retrospective studies that compared the effects of RYGB, SG, and OAGB on T2DM remission. Studies published between 2001 and 2022 were analysed. Only patients with T2DM and who had primary bariatric surgery were included. *Results*: After applying the inclusion and exclusion criteria, seven articles were included in the review. It was found that all three procedures had comparable T2DM remission. RYGB was noted to have the highest complication rate when compared to SG and OAGB. Importantly, it was noted that other predictive factors such as age, duration of diabetes, baseline HbA1c, BMI, and use of antidiabetic medication play a crucial role in T2DM remission. *Conclusions*: This systematic literature review confirms the existing data that all three bariatric surgeries induce remission of T2DM. Increasing in popularity, OAGB had comparable outcomes to RYGB and SG in inducing T2DM remission. In addition to the choice of bariatric surgery, there are other independent predictive factors that have an impact on T2DM remission. Further studies with larger sample sizes, longer follow-up periods, and studies that control confounding factors are required in this field.

## 1. Introduction

More than one in four adults in the United Kingdom are classified as obese, and nearly two-thirds are classified as overweight [1]. Furthermore, obesity accounts for 80–85% of the risk of developing type 2 diabetes [1]. Type 2 diabetes mellitus (T2DM) is known to have substantial psychological consequences on patients. It is associated with greater levels of depression and anxiety and can interfere with social and occupational activities, all leading to reduced quality of life in patients [2]. In turn, this translates to a substantial socioeconomic burden. Diabetes-related complications such as retinopathy, nephropathy, and metabolic disease are all known to be associated with poorer quality of life. Compared with lifestyle modifications and medical management, emerging evidence suggests that bariatric surgery is a superior treatment for diabetes in severely obese patients [3]. Perioperative mortality is very low, ranging between 0.03% and 0.2% [4]. Thus, it is not surprising that metabolic surgery has become one of the most commonly performed operations in general surgery [4]. Furthermore, the data show marked improvement in a range of obesity-related diseases postoperatively. Of significance is the reduction in the prevalence of T2DM from 30% to 14% one year post-surgery [5].

The choice of bariatric procedure primarily lies with the patient, although the surgeon also plays an important role in guiding the patient to choose the procedure that will most benefit them. Factors such as underlying comorbidities, success rate, complications and invasiveness of procedure should be taken into consideration [6]. The benefits of bariatric surgery with regard to remission of T2DM are multifactorial (decreased insulin resistance, increase in circulating bile acids leading to increased insulin sensitivity, and an upregulation of GLP-1 leading to the stimulation of pancreatic β cells) and actually extend beyond weight loss [7]. Though bariatric surgery was traditionally intended as a treatment for weight loss, it has demonstrated major effects on reducing rates of T2DM, improving cardiovascular health and, in turn, reducing morbidity and mortality [8]. The latest recommendation by the American Diabetes Association defines diabetes remission as “a return of HbA1c to less than 6.5% or 48 mmol/mol that occurs spontaneously or following an intervention and persists for at least 3 months in the absence of usual glucose-lowering pharmacotherapy” [9].

Of the limited studies comparing the effectiveness of bariatric surgery on the remission of T2DM, this systematic review aims to analyse and compare the findings of currently available studies on the short-term and long-term remission of T2DM after undergoing one of the three bariatric surgeries.

## 2. Materials and Methods

This systematic literature review was performed in keeping with the Preferred Reporting Items for Systematic Reviews and Meta-Analyses (PRISMA) statement [10]. The protocol of this systematic review was not registered in advanced. Articles were obtained from PubMed, EMBASE, and COCHRANE. The keywords used to search include “Type 2 Diabetes Mellitus”, “Bariatric surgery”, “Sleeve gastrectomy”, “Roux-en-Y Gastric Bypass”, “One-anastomosis Gastric Bypass”, “Mini-Gastric Bypass”, and “Remission”. A filter was applied to only include articles with full text and published in the last twenty-one years (from 2001–2022). The inclusion criteria were studies that included patients diagnosed with T2DM based on medical history, HbA1c ≥ 6.5% or a previous fasting blood glucose > 7.0 mmol/L, and who underwent primary bariatric surgery. Only articles published in English and comparing all three bariatric procedures; Roux-en-Y gastric bypass, sleeve gastrectomy, and one-anastomosis gastric bypass were included. Articles included were prospective studies, retrospective studies, and randomised control trials. Studies that looked at prediabetes, type 1 diabetes mellitus, or revisional bariatric surgeries were excluded. Furthermore, studies that did not compare the three bariatric surgeries were excluded. Published articles where the full text was not available were also excluded.

The quality of studies was rated using the Newcastle–Ottawa scale (NOS) for non-randomised trials [11] (Table 1). The NOS has three domains and assigns up to a maximum of nine points for the highest quality studies: (1) selection of study groups (4 points), (2) comparability of groups (2 points), and (3) ascertainment of exposure and outcomes (3 points) for cohort studies.

## 3. Results

**Figure 1:**
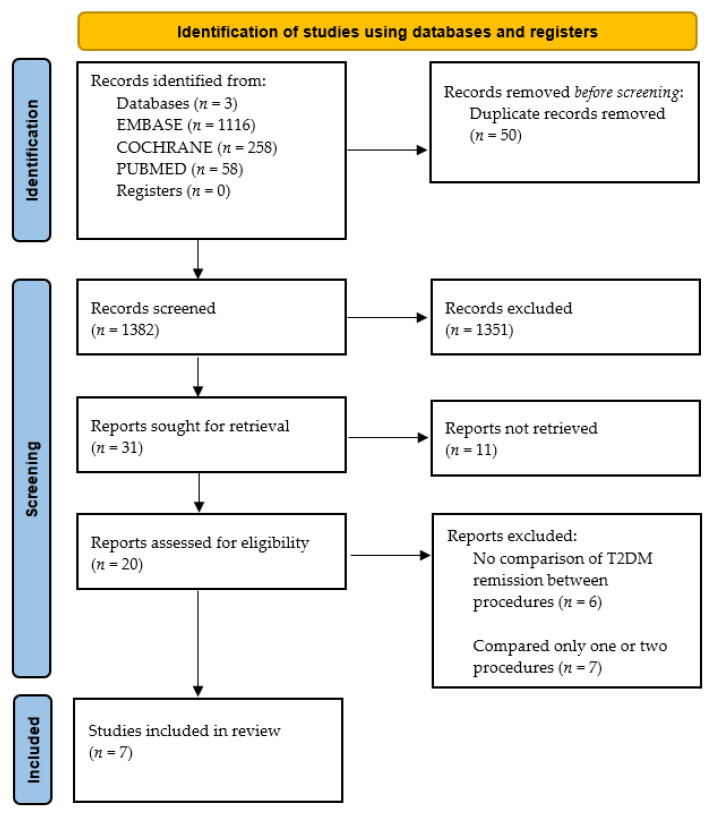
PRISMA Flowchart.

A total of 1432 (Embase 1116, PubMed 58, Cochrane 258) records were identified. After removing 50 duplicates, 1382 papers were screened using the title and abstract. One thousand one hundred and fifty-one articles were excluded as they (1) did not investigate T2DM remission, (2) only studied one or two of the three bariatric procedures or included revisional procedures, (3) were comments/letters/systematic reviews/meta-analysis/conference abstracts, and/or (4) were not in English. A total of 20 full papers were read. Thirteen were excluded as they did not compare T2DM remission after bariatric surgery (*n* = 6) or compared only one or two rather than all three bariatric procedures (*n* = 7). Finally, seven articles were included in this literature review (Figure 1). The baseline characteristics for each article are described in Table 2.

Castro et al. [12] conducted a retrospective study to compare and analyse the long-term weight loss, nutritional deficiencies, and remission of comorbidities for patients who underwent RYGB, OAGB, and SG. Remission of T2DM was defined as plasma glucose < 5.55 mmol/L or HbA1c < 6% in the absence of hypoglycaemic treatment. A total of 358 patients were included in the study, with a mean BMI of 44 ± 9.1 kg/m^2^. All patients were on oral antidiabetic agents, and 23.2% were using insulin. The remission of T2DM was highest in the OAGB group (91.9%) compared to the RYGB (83.6%) and SG (81.9%) one year postoperative (*p* = 0.038). At two years postoperative, the remission of T2DM was OAGB (91.9%), RYGB (83.6%), SG (79.5%) (*p* = 0.018). At five years postoperative, the remission rate was OAGB (89.4%), RYGB (80.3%), SG (75.9%) (*p* = 0.029). The study then compared the rate of remission in patients who were on insulin treatment versus those who were not. It was found that patients who were in the non-insulin group had a higher remission rate across all their intervention groups, and it was statistically significant in the RYGB and OAGB groups (*p* = 0.001, *p* = 0.019), respectively. The duration of T2DM was not associated with complete remission in any of the groups. Additionally, BMI was significantly lower, and excess BMI loss was markedly higher after OAGB compared to RYGB and SG at one, two, and five years after surgery (*p* < 0.001).

Soong et al. [13] conducted a study to compare the safety and efficacy of RYGB, OAGB, and SG in superobese patients (BMI > 50 kg/m^2^). This was a retrospective study, and a total of 498 patients’ data (62 RYGB, 190 SG, and 246 OAGB) were recruited for the study. T2DM remission was defined as HbA1c < 6.5% without medication. With regard to remission of T2DM, patients who underwent RYGB, OAGB, and SG achieved a 100% remission rate at five years post-surgery, and there was no significant difference between the groups. At five years, patients who underwent OAGB had a higher total weight loss (TWL) percentage (40.8%) compared to RYGB (37.2%, *p* = 0.130) and SG (35.1%, *p* = 0.005). OAGB (64.5%, 68.0%, and 67.7%) also showed a higher EWL percentage at one, five, and ten years compared to RYGB (62.4%, 60.8%, and 56.4%) and SG (59.5%, 60.3%, and 52.3%), respectively.

Toh et al. [14] conducted a 5-year retrospective observational study to compare the effects of RYGB, SG, and OAGB. The primary endpoint was excess weight loss and T2DM remission, while the secondary endpoint was postoperative complications. T2DM remission was defined as HbA1c < 6.5% without antidiabetic medications at one year post-surgery. It was found that the remission rate for T2DM was high in all three groups, RYGB (86.9%), OAGB (71.9%), SG (82.2%), at one year post-operation. There was no significant difference between the groups (*p* = 0.162). The EWL at six months was highest for the RYGB (60.2%) and OAGB (58%) groups compared to the SG (49.7%) group. At years one and two, the EWL was comparable among all three groups, whereas at years three, four, and five, RYGB (50.1%, 48.7%, and 47.7%, respectively) and OAGB (66.2%, 64%, and 65.2%, respectively) showed significantly higher EWL compared to the SG group (47.8%, 40.8%, and 47.3%, respectively). After adjusting for confounding factors, over the 5-year period, RYGB and OAGB had significantly higher EWL compared to the SG group (*p* = 0.027 and *p* = 0.006), respectively. Moreover, the EWL was highest between six months to three years, then slowed down at years four and five. The overall complication rate for all three procedures was low at 3.7%, and it was significantly lower in the SG group than in the RYGB and OAGB groups (*p* = 0.003).

Moradi et al. [15] conducted a retrospective study on obese patients undergoing OAGB, RYGB, and SG to determine the rate of T2DM at one year and three years after bariatric surgery and the characteristics that help predict the remission of T2DM post-surgery. T2DM remission was categorised into complete remission (HbA1c < 6% and FBG < 100 mg/dL without the use of antidiabetic medication) and partial remission (HbA1c 6–6.4% and FBG 100–125 mg/dL without the use of antidiabetic medication). A total of 1351 patients with T2DM were analysed (675 OAGB, 475 RYGB, and 201 SG). There was a significant overall reduction in BMI, FBG, HbA1C, insulin use, and cessation of oral antidiabetic medications at year one and year three post-operation. Of the total, 74.8% of patients achieved complete diabetes remission at year one and maintained at 79.4% in year three. There was no statistical significance between the three procedures at year one and year three RYGB (75.6%, 79.4%), OAGB (75.4%, 80.4%), and SG (70.6%, 75.8%), respectively. At year one, three patients who underwent OAGB had a recurrence of T2DM compared to zero for the RYGB and OAGB group. At year three, the number of patients who had recurrence increased in all three procedures: RYGB by 4, OAGB by 9, and SG by 4.

Tabesh et al. [16] conducted a retrospective study on 485 patients (SG 50.3%, RYGB 19.6%, OAGB 30.1%) and had a follow-up period of one year. The aim of the study was to assess the effects of the three bariatric procedures on cardiometabolic risk factors. T2DM was defined as having a FBG > 126 mg/dL. At baseline, 103 patients had T2DM and there was no significant difference between the groups. Overall, there was a significant improvement in the number of patients with T2DM in one year, from 103 to 3 (*p* < 0.001). When comparing between the three procedures, there was no significant difference among the groups. Moreover, patients undergoing RYGB and OAGB had significantly reduced body weight, fat mass (FM), BMI, and fat mass-to-fat-free mass ratio compared with SG (*p* < 0.05). Patients who underwent RYGB also had significantly higher TWL percentage compared to the SG and OAGB group.

Wasir et al. [17] conducted a retrospective observational study on 121 patients (14.87% SG, 64.46% RYGB, 1.65% OAGB, and 19% AGB) who underwent bariatric surgery. Remission of T2DM was defined as FPG < 7 mmol/L or HbA1C < 6.5% without antidiabetic medication. A total of 68.6% of patients achieved T2DM remission at two years post-surgery. Patients who underwent RYGB: 80.8% (*n* = 63) had the highest remission rate, followed by SG: 55.6% (*n* = 10), and OAGB: 50% (*n* = 1). The mean duration of diabetes preoperation for patients who achieved remission was significantly shorter (4.38 ± 3.87 years) than those with a longer duration of 9.41 ± 3.94 years (*p* < 0.0001). Moreover, patients who achieved T2DM remission had a significantly greater mean percentage weight loss (29.55 ± 10.44%) compared to patients who did not achieve remission 20.52 ± 10.79% (*p* < 0.001). The mean preoperative HbA1c for patients who achieved remission was significantly lower (56.97 ± 14.82 mmol/mol) compared to those who did not achieve remission (70.65 ± 16.25 mmol/mol) (*p* < 0.001).

Shen et al. [18] conducted a study on 1016 patients who underwent five bariatric surgeries (197 RYGBs, 130 SG-DJBs, 171 OAGBs, 81 SA-DJBSGs, and 437 SGs). The primary outcome was glucose control one year after bariatric surgery. Complete remission of T2DM was defined as HbA1c < 6.0% without antidiabetic medication and partial remission was defined as HbA1c < 6.5% without antidiabetic medication. Patients in the OAGB group showed a statistically significant higher complete remission rate (78.4%) than did the other groups (*p* = 0.02). SG had a complete remission rate of 69.8%, followed by RYGB at 65%. The procedure with the least effect on remission rates were SG-DJB and SA-DJBSG. Moreover, patients undergoing OAGB had the highest %TWL of 30.5% compared to the other procedures (*p* < 0.001).

## 4. Discussion

The latest guidelines by the American Society for Metabolic and Bariatric Surgery (ASMBS) and the International Federation for the Surgery of Obesity and Metabolic Disorders (IFSO) include [4] (1) “Metabolic and bariatric surgery (MBS) is recommended for individuals with a body mass index (BMI) of 35 kg/m^2^, regardless of the presence, absence, or severity of co-morbidities”; (2) “MBS should be considered for individuals with metabolic disease and BMI of 30–34.9 kg/m^2^”; (3) “BMI thresholds should be adjusted in the Asian population such that a BMI 25 kg/m^2^ suggests clinical obesity, and individuals with BMI 27.5 kg/m^2^ should be offered MBS”. The Third National Bariatric Surgery Registry report shows that Roux-en-Y gastric bypass (RYGB) remains the most commonly performed bariatric operation within the United Kingdom (49%), followed by sleeve gastrectomy (SG) (35%), whilst one-anastomosis gastric bypass (OAGB) accounts for 9% of surgeries [5]. With the rapid development of bariatric surgery, OAGB is becoming the treatment of choice for obese patients after RYGB and SG [19]. Laparoscopic sleeve gastrectomy (LSG) is the most common bariatric and metabolic surgery procedure, accounting for up to 67% of all bariatric procedures globally and in the US [19]. These surgeries have different techniques for inducing weight loss and remission of comorbidities of obesity, such as T2DM, hypertensive disease, and hyperlipidaemia, using restrictive and/or malabsorptive measures. Most of the studies available compare the outcomes of two surgeries; however, there is limited literature that compares and analyses the outcomes of the three most common bariatric surgeries (RYGB, SG, OAGB) with regard to T2DM remission. Hence, this systematic review aims to analyse the outcomes of the three most common bariatric surgeries.

In addition to understanding the pathophysiology of T2DM remission after bariatric surgery, it is worth noting that there are scoring systems and predictive factors that are used to guide the selection of bariatric surgery. The three common scoring tools are the ABCD tool, DiaRem, and advanced DiaRem (Ad-DiaRem). The ABCD diabetes surgery score considers the patient’s age, BMI, C-peptide level, and duration of T2DM in years [20]. DiaRem takes into account the patient’s age, HbA1c, use of insulin treatment and type of antidiabetic drugs used [21]. Ad-DiaRem includes the same components of the DiaRem score, with the addition of diabetes duration in years and the number of antidiabetic drugs used [22]. Gupta et al. [23] conducted a retrospective study on a cohort of Indian patients in a tertiary centre who underwent bariatric surgery (RYGB, OAGB, or SG) to study the correlation of three preoperative scores (ABCD, DiaRem, and Ad-DiaRem) and the percentage of T2DM remission achieved. The cutoff scores for predicting diabetes remission for DiaRem, Ad-DiaRem, and ABCD score were <7, <10, and >6, respectively. At 36 months, the overall remission rate (complete and partial remission) was 55.2%. It was found that the duration of T2DM pre-operation reflected a significant difference with regard to patients achieving remission (median value: 18 months) and non-remission (median value: 90 months) (*p* < 0.01). Patients in the remission group also had significantly lower HbA1c levels and higher C-peptide levels, and fewer patients required insulin (*p* < 0.01). In general, higher C-peptide levels suggest that there has not yet been significant damage to the pancreas; thus, surgical intervention can still play a role in the remission of diabetes. The Ad-DiaRem score showed the highest predictive accuracy (81.1%) of patients achieving T2DM remission compared to the DiaRem (75.6%) and ABCD score (77.8%). The positive and negative predictive values for the three scores were DiaRem (89.5% and 65.4%, respectively), ABCD score (86.7% and 66.7%, respectively), and Ad-DiaRem score (83.0% and 78.4%, respectively).

Aminian et al. [24] conducted a study to construct and validate a scoring system to aid in the selection of bariatric surgery procedures according to the severity of T2DM. The mean follow-up period was seven years (range 5–12). Patients were classified into three severity groups: mild, moderate, and severe. This classification was based on preoperative HbA1c, duration of T2DM, number of diabetes medications, and insulin use before surgery. This study compared RYGB with SG. It found that patients in the mild and moderate group who underwent RYGB showed a statistically significant higher remission percentage than the SG group (*p* = 0.04 and *p* < 0.0001). There was no significant difference in remission among the bariatric procedures in the severe group. Compared to the findings from previous studies, this highlights that other independent factors, such as the patient’s preoperative diabetes status and severity contribute to the remission rate, and remission is not solely based on the type of bariatric procedure performed.

Moreover, Dang et al. [25] conducted a study looking at the predictive factors for diabetes remission in patients undergoing laparoscopic SG and laparoscopic RYGB. Patients with diabetes remission were younger (*p* = 0.004), had a shorter duration of diabetes preoperatively (*p* < 0.001), used less preoperative insulin (*p* < 0.001), had fewer oral hypoglycaemic medications (*p* < 0.001), and also had lower HbA1c (*p* < 0.001). The duration of diabetes does play a role, as it is a reflection of the residual B-cell mass in patients with T2DM.

Hence, it is evident that parameters such as the use of insulin, duration of diabetes, patient’s age, pre-operation BMI, HbA1c, and C-peptide levels play a role in T2DM remission. This is in keeping with the study by Moradi et al. [15] that showed, after carrying out a multivariable logistic regression model, patients who are younger, not on insulin therapy, lower FBG and HbAIc pre-op, had shorter duration of T2DM pre-op, and do not have a family history of obesity had significantly higher chances of diabetes remission at year one and year three after surgery (*p* < 0.05). At year one, the rate of complete remission of T2DM was higher in the RYGB and OAGB group compared to SG group; however, it was not statistically significant. There was a 0.22% of recurrence of T2DM at one year and 1.99% at three years. One of the strengths of this study is the large sample size and good follow-up sample of patients at year one (1351) and year three (853). Aditionally, potential confounding factors were adjusted when comparing the three procedures.

Shen et al. [18] used the ABCD score to quantify the severity of T2DM in patients undergoing bariatric surgery. Patients who underwent SG had the highest (6.5 ± 2.0) score, followed by OAGB (5.2 ± 2.2) and RYGB (5.2 ± 2.3), with the SG-DJB group having the lowest (4.6 ± 2.0) score (*p* < 0.05). After stratifying the procedures based on scores, patients with higher ABCD scores had higher remission rates. As the ABCD score plays a role in the remission rate of T2DM [23], categorising bariatric procedures into scores would help minimise other confounding factors and consider the procedure’s efficacy in inducing T2DM remission. In the subgroup of 10–9, patients who underwent OAGB and RYGB had 100% complete remission, and SG had 81.8% remission; however, it was not statistically significant. There was no statistical significance between the three procedures in patients with lower scores.

Wasir et al. [17] noted higher remission rates in patients who were on antidiabetic medications (81.9%) compared to those on insulin (39.5%). Additionally, patients who achieved T2DM had a shorter duration of diabetes pre-operation and a lower pre-operation HbA1c. In patients who achieved remission, there was a significantly higher percentage of weight loss. Interestingly, there was no statistical difference in baseline BMI among patients who achieved remission and those who did not. Although RYGB had the highest remission rate, followed by SG and OAGB, factors such as pre-operation HbA1c levels, duration of diabetes, and use of antidiabetic medications for each of the three procedures were not mentioned, and this could affect the outcome of T2DM remission.

The study by Castro et al. [12] showed that EBMIL was significantly higher in the OAGB group compared to the RYGB and SG groups at years one, two, and five. Moreover, the EBMIL increased from year one to year two, and then reduced at year five. The weight curve showed weight regain at year five post-surgery. Moreover, there was statistical significance in the percentage of T2DM remission across all three interventions. When comparing intervention groups, the OAGB group showed significantly higher rates of T2DM remission than the SG group in years one, two, and five. There was no significant difference between RYGB versus SG and RYGB versus OAGB. There was also no significant difference in baseline age, sex, BMI, duration of diabetes, use of insulin, and anti-diabetic medication for all three groups. Castro et al. [12] went on to compare patients who used insulin and those who did not use insulin. It was found that patients who did not use insulin showed a higher remission rate of T2DM across all three operations than those who used insulin. This was statistically significant in the RYGB and OAGB groups. Further analysis of the insulin group showed no difference in remission across the three intervention groups. This indicates that the use of insulin pre-operatively impacts T2DM remission. Interestingly, there was no significant association between the duration of T2DM before surgery and complete remission in all three groups. This finding is not consistent with the findings by Dang et al. [25], which showed that the duration of T2DM pre-surgery impacts the remission of T2DM. One of the strengths of this study was that a comparison was made between patients who used insulin versus patients who did not use insulin and it showed the effects of preoperative insulin on T2DM remission. Moreover, other strengths include the long follow-up period of five years.

Interestingly, the study by Toh et al. [14] showed a different finding compared to the study carried out by Castro et al. [12], whereby there are high rates (ranging from 72–87%) of T2DM remission at one year post-surgery for all three groups. There was no statistical significance between the groups (*p* = 0.179). This could be due to the fact that the baseline HbA1c levels in the LSG group were lower (6.4%) compared to OAGB (8.2%) and RYGB (7.8%) groups. Moreover, Toh et al. [14] showed that, across all three intervention groups, while the percentage of EWL was comparable at years one and two, for years three, four, and five, RYGB and OAGB had significantly higher EWL than SG. These findings were similar to the study carried out by Currie et al. [26]. Additionally, the EWL was highest between six months to three years, then gradually reduced at years four and five. One of the strengths of this study is that comparisons were made between different ethnicities within an Asian population. Limitations of this study include the short follow-up period for T2DM remission (one year), and a high percentage of original participants lost to follow-up (46.8%).

Similar to the findings by Toh et al. [14], Tabesh et al. [16] also showed a significantly high T2DM remission at one year for all three procedures; however, there was no significant difference between the groups. Moreover, the study showed a correlation between a high % TWL and FM, TG, and FBG. One of the strengths of this study is the fact that the patients did not take antidiabetic medications, which could affect the outcome of T2DM remission after surgery. On the other hand, this could potentially be the reason for the significant drop in T2DM. One of the weaknesses of the study is the lack of data on HbA1c levels, duration of T2DM, and the other predictors for remission. Additionally, patients were not randomised into groups, and this can lead to selection bias.

Unlike the other studies analysed, the study carried out by Soong et al. [13] was unique, as it looked into superobese patients (BMI > 50) undergoing bariatric surgery, and there was a long follow-up period (ten years). Pre-operation BMI is one of the four components of the ABCD scoring tool, and patients with a BMI > 50 were assigned a score of three for that component [20]. The mean BMI was 56.0 ± 6.7 kg/m^2^, and there was no significant difference in the baseline age, BMI, and HbA1c for all three groups. At five years post-surgery, patients who underwent RYGB, OAGB, and SG showed 100% remission of T2DM. The rates of T2DM remission before five years and at ten years were not reported. Interestingly, patients who underwent OAGB also showed a sustained weight loss of up to ten years compared to the RYGB and SG groups. The safety profile for patients undergoing OAGB and SG was similar. Both procedures had fewer complications than the RYGB group. This study shows that superobese patients benefit from any of the three procedures with regard to T2DM remission. Moreover, when considering the safety profile, revision rate, and overall weight loss in superobese patients, OAGB and SG are more favourable than RYGB. Further studies comparing obese with super obese patients should be carried out. One of the limitations of this study was the surgical technique whereby a longer-than-average bypass limb was used, which could account for the higher complication rate. Moreover, it was reported that there was an overall revision rate of 5.4% for the whole group. The revision of surgery could affect the remission rate of T2DM.

Besides looking at the success rate for T2DM remission, it is also worth noting some of the possible complications that could arise from these operations. It is evident that out of the three procedures, RYGB is the most complex. Some of the advantages of SG compared to RYGB include its technical simplicity, lack of anastomosis, the reduced extent of resection, and shorter operative time [6]. Furthermore, OAGB has the added benefit compared with RYGB as it avoids a tension-free gastrojejunal anastomosis, as well as a Roux-en-Y limb construction and its complications [27].

A study by Soong et al. [13] found that the overall complication rate for patients who underwent RYGB was 17.7%. RYGB also had a statistically significant major complication rate (4.8%) compared to OAGB (0.8%) and SG (0.5%), and there was no difference between the SG and OAGB groups. The revision rates for SG, RYGB, and OAGB were 2.6% (5/190), 8.1% (5/62), and 6.9% (17/246), respectively. The most common cause for revision in the OAGB and RYGB groups was malnutrition. A study carried out by Melissas et al. [28] found that the early (<30 days) postoperative complication rate after RYGB was significantly higher (3.02%) compared to SG (2.12%) (*p* = 0.0006). The postoperative leak rate was also significantly higher in the RYGB group. Bleeding was the most common complication for both groups. The rate of late (>30 days) postoperative complications requiring hospitalisation was higher in the RYGB group (3.30%) compared to the SG group (0.07%) (*p* < 0.0001). The most common late complications developed were intestinal obstruction and anastomotic ulcers. Docimo et al. [29] found that the 30-day rate of mortality of patients (0.36%, 0.36%), readmission (3.23%, 5.02%), reoperation (1.79%, 3.23%), adverse event (7.88%, 8.96%), anastomotic leak (1.79%, 1.08%), and reintervention (1.79%, 2.51%) were not significantly different between patients undergoing RYGB and OAGB, respectively. The most common causes of readmission in OAGB patients were nausea, vomiting, and nutritional depletion.

Overall, all three procedures have their advantages and disadvantages. The recent report by IFSO showed preferential use of RYGB and OAGB compared to SG in the choice of bariatric surgery for T2DM control [30]. More trials with a large sample size that directly compares all three procedures are needed to get a better understanding of the role of each procedure with regard to T2DM remission. Moreover, information such as duration of diabetes, the use of insulin/anti-diabetic agents, BMI, age, HbA1c, and C-peptide levels should be recorded to obtain a better comparison among procedures after taking into account predictive factors.

One of the limitations of this review is the narrow inclusion criteria, where only studies that compared all three bariatric procedures were included. Additionally, there was a lack of a standardised definition for short-term, medium-term, and long-term in all the studies. This can cause confusion and pose a problem for narrative and systematic reviews. Mahawar [31] proposed the following terminologies: short-term (<1 year), medium-term (1–5 years), long-term (5–10 years), and very long-term (>10 years). By utilising standardised terminology that is widely accepted, results would be more generalisable when comparing and evaluating the outcomes of bariatric surgery in the short-term, medium-term, and long-term. This would also allow clinicians and patients to be aware of the best period to appreciate the benefits of bariatric surgery in inducing T2DM, and to be mindful of when the benefits are likely to slow down or diminish, hence paying extra attention to ensure the disease does not relapse. For instance, a study by Mingrone et al. [32] showed that 44% of patients who underwent RYGB or biliopancreatic diversion surgery had hyperglycaemia relapse after two years.

Furthermore, another limitation was the varying definitions of T2DM remission. In 2009, the American Diabetes Association (ADA) reached a consensus and suggested that T2DM remission is categorised into partial or complete remission [33]. Partial remission was defined as an HbA1c level below <48 mmol/mol (6.5%) and/or fasting glucose 5.6–6.9 mmol/L of at least one year duration in the absence of pharmacotherapy agents. Complete remission was defined as a return to normal levels, where the HbA1c level was within normal limits and fasting glucose < 5.6 mmol/L for at least one year in the absence of pharmacotherapy agents. However, the latest consensus by the American Diabetes Association defined remission as “a return of HbA1c to less than 6.5% or 48 mmol/mol that occurs spontaneously or following an intervention and persists for at least three months in the absence of usual glucose-lowering pharmacotherapy” [9]. The change in definition makes it challenging to compare the outcomes of T2DM remission between older and newer studies.

## 5. Conclusions

This systematic literature review highlights the current evidence of the effects of sleeve gastrectomy, one-anastomosis gastric bypass, and Roux-en-Y gastric bypass on the remission of T2DM. It is evident that all three of these surgeries have the potential to induce remission of T2DM. Factors such as age, baseline BMI, HbA1c, use of antidiabetic medication and duration of diabetes plays a major role in T2DM remission alongside the choice of bariatric surgery. OAGB, being the latest bariatric surgery, is gaining popularity among bariatric surgeons worldwide and has comparable results in inducing weight loss and T2DM remission. With regards to complication rate, RYGB has the highest complication rate and SG has the lowest. Hence, it is worth noting that none of the three surgeries offers an evident superior outcome compared to the other with regard to T2DM remission. T2DM remission post-bariatric surgery involves a complex physiology, and the patients’ baseline characteristic should be taken into consideration.

## Figures and Tables

**Table 1:** Quality assessment by the Newcastle–Ottawa Scale.

Study	S1	S2	S3	S4	C1	O1	O2	O3	Total
Castro et al. (2020) [12]	*	*	*	*	**	*	*	*	9
Soong et al. (2021) [13]	*	*	*	*	*	*	*	-	7
Toh et al. (2018) [14]	*	*	*	*	*	*	*	-	7
Moradi et al. (2022) [15]	*	*	*	*	**	*	*	*	9
Tabesh et al. (2022) [16]	*	*	*	*	*	*	*	*	8
Wasir et al. (2019) [17]	*	*	*	*	-	*	*	*	7
Shen et al. (2021) [18]	*	*	*	*	*	*	*	*	8

S1: representativeness, S2: selection, S3: ascertainment, S4: demonstration, C1: comparability, O1: outcome selection, O2: outcome follow-up, O3: adequacy. Maximum score for S1–S4 and O1–O3 is 1 star. Maximum score for C1 is 2 stars. * The study suffices in this criterion point. ** The study suffices in these two criteria points.

**Table 2:** Baseline characteristics.

Study	Population (*n*)	Age (Years)	BMI (kg/m^2^)	Study Design	Follow up (Months)	T2DM Variables	Population with T2DM	Duration of T2DM (Years)	Insulin Treatment (*n*)	HbA1c (%)	Remission Rate RYGB (%)	Remission Rate OAGB (%)	Remission Rate SG (%)
Toh et al. (2018) [14]	561	43.6	41.4	Retrospective	12	HbA1C	189	-	-	7.5	86.9	71.9	82.2
Wasir et al. (2019) [17]	121	48.2	49.8	Retrospective	24	FPG, HbA1C	121	5.98	38	7.8	80.8	50%	55.6
Castro et al. (2020) [12]	358	43.3	44.0	Retrospective	12, 24, 60	BG, HbA1C	358	6.3	28	5.8	12–83.6	12–91.9	12–81.9
											24–83.6	24–91.9	24–79.5
											60–80.3	60–89.4	60–75.9
Shen et al. (2021) [18]	1016	42.9	39	Retrospective	12	FBG, HbA1C	1016	5	220	8.3	65.0	78.4	69.8
Soong et al. (2021) [13]	498	32.1	56.0	Retrospective	60	BG, HbA1C	219	-	-	6.5	100	100	100
Moradi et al. (2022) [15]	1351	47.3	44.1	Retrospective	12, 36	FBG, HbA1C	1351	5	385	7.8	12–75.6	12–75.4	12–70.6
											36–79.4	36–80.4	36–75.8
Tabesh et al. (2022) [16]	485	41.6	46.1	Retrospective	12	FBG,	103	-	0	-	100	98.6	99.6

BMI (body mass index), HbA1c (haemoglobin A1c), RYGB (Roux-en-Y gastric bypass), OAGB (one-anastomosis gastric bypass), SG (sleeve gastrectomy).

## Data Availability

Data will be made accessible upon reasonable request. Please email the corresponding author.
